# Comparative Lipidomics of Oral Commensal and Opportunistic Bacteria

**DOI:** 10.3390/metabo14040240

**Published:** 2024-04-20

**Authors:** Paul L. Wood, Annie Le, Dominic L. Palazzolo

**Affiliations:** 1 Metabolomics Unit, College of Veterinary Medicine, Lincoln Memorial University, 6965 Cumberland Gap Pkwy., Harrogate, TN 37752, USA; 2 Clinical Training Program, DeBusk College of Osteopathic Medicine, Lincoln Memorial University, 6965 Cumberland Gap Pkwy., Harrogate, TN 37752, USA; 3 Department of Physiology, DeBusk College of Osteopathic Medicine, Lincoln Memorial University, 6965 Cumberland Gap Pkwy., Harrogate, TN 37752, USA; domenico.palazzolo@lmunet.edu

**Keywords:** oral bacterial lipidomics, high-resolution mass spectrometry

## Abstract

The oral cavity contains a vast array of microbes that contribute to the balance between oral health and disease. In addition, oral bacteria can gain access to the circulation and contribute to other diseases and chronic conditions. There are a limited number of publications available regarding the comparative lipidomics of oral bacteria and fungi involved in the construction of oral biofilms, hence our decision to study the lipidomics of representative oral bacteria and a fungus. We performed high-resolution mass spectrometric analyses (<2.0 ppm mass error) of the lipidomes from five Gram-positive commensal bacteria: *Streptococcus oralis*, *Streptococcus intermedius*, *Streptococcus mitis*, *Streptococcus sanguinis*, and *Streptococcus gordonii*; five Gram-positive opportunistic bacteria: *Streptococcus mutans*, *Staphylococcus epidermis*, *Streptococcus acidominimus*, *Actinomyces viscosus*, and *Nanosynbacter lyticus*; seven Gram-negative opportunistic bacteria: *Porphyromonas gingivalis. Prevotella brevis*, *Proteus vulgaris*, *Fusobacterium nucleatum*, *Veillonella parvula*, *Treponema denticola*, and *Alkermansia muciniphila*; and one fungus: *Candida albicans*. Our mass spectrometric analytical platform allowed for a detailed evaluation of the many structural modifications made by microbes for the three major lipid scaffolds: glycerol, sphingosine and fatty acyls of hydroxy fatty acids (FAHFAs).

## 1. Introduction

Omics technologies, particularly genomics, are increasing our knowledge base of microbial communities resident in oral and gastrointestinal compartments. This information increases our understanding of the pathogenic processes that contribute to a diversity of oral diseases. Within the oral cavity, microbes are prevalent on the tongue, buccal mucosa, palate, gingiva, teeth, salivary glands, and saliva. Diverse microbial populations of over 600 bacterial species and 100 fungal species have been identified [1], encompassing both commensal and opportunistic bacteria and fungi.

Common commensal bacteria, such as *Streptococcus intermedius*, *Streptococcus mitis*, *Streptococcus sanguinis* and *Streptococcus gordonii*, are known as early colonizers that adhere to the underlying epithelial cells and function as a protective barrier [2]. They serve as a scaffold for other oral bacteria, ultimately leading to the formation of multi-species biofilms [3]. These commensal species are in symbiosis with their human hosts, antagonizing the growth of opportunistic bacteria [4], thereby aiding in the prevention of dental carries and periodontal disease [5].

With regard to oral diseases, tooth decay (enamel destruction) and periodontitis (plaque formation and gum weakening) are caused by bacteria. *Streptococcus mutans* is a key player in tooth decay while *Porphyromonas gingivalis* and *Fusobacterium nucleatum* are involved in periodontitis. *F. nucleatum* is unique in that it is a bridge that links early and late bacterial colonizers involved in plaque biofilm formation [6]. The mechanisms of polymicrobial biofilm formation and pathogenicity involve complex membrane protein–lipid–carbohydrate complexes [7] generated by multiple interacting bacterial and fungal species [8,9,10]. For example, in bacterial interactions, the growth of *Veillonella parvula* is augmented by the supply of lactic acid provided by *Streptococcus* species [9], while in fungal–bacterial interactions, polysaccharides secreted by *Candida albicans* augment *Streptococcus mutans*’ contribution to biofilm formation [10,11]. Fungal–bacterial interactions result in increased virulence, as evidenced by invasive candidiasis and early childhood caries with *C. albicans* combined with *Streptococcus* spp. [10,11].

Oral health and overall systemic health are intrinsically linked. Oral opportunistic bacteria can also enter the bloodstream and result in systemic infections and infections of other tissues. Current thoughts are that oral bacteria can augment inflammatory processes involved in cardiovascular disease [6], respiratory disease [12], rheumatoid arthritis [13], cancer [1,14,15], Alzheimer’s disease [16], multiple sclerosis [8], and bacterial vaginosis associated with poor pregnancy outcomes [17,18,19]. For example, *P. gingivalis* is linked to diabetes, cardiovascular diseases and Alzheimer’s disease [16]. Similarly, several species of oral commensal streptococci, including *S. gordonii*, *S. mitis*, *S. sanguinis* and *S*. *oralis*, have also been implicated in infective endocarditis [7]. From these reports, it is evident that a healthy balance of both commensal and opportunistic microbes in biofilms of multi-species communities is required to maintain not only good oral health, but ultimately systemic health. The lipodomic profiles associated with these microbes provide for proper construction of oral biofilms [9,10].

These lipodomic profiles associated with oral microbes are intimately involved with the formation of these biofilms. To better understand how they are constructed, it is imperative that lipid profiles for oral bacteria and fungi be defined. However, at this time lipidomics evaluations of oral bacteria and fungi have been limited to *T. denticola* [20], *S. mutans* [21,22,23], and *C. albicans* [24,25]. We undertook a larger comparative study utilizing electrospray high resolution mass spectrometry (ESI-HRMS) to monitor lipids and to validate their identity by MS^2^. ESI-HRMS provides the sensitivity and specificity required for the analyses of diverse complex bacterial lipids that possess aminoacyl, peptidyl, and glycosyl modifications [26,27].

## 2. Materials and Methods

### 2.1. Bacterial Processing

All reagents and supplies were purchased from ThermoFisher Scientific (Waltham, MA, USA) unless otherwise indicated. Bacterial pellets (Table 1), purchased from the American Type Culture Collection (ATTC) (Manassas, VA, USA), were sonicated (Thermo Fisher FB50, Waltham, MA, USA) in 1 mL of methanol and 1 mL of water containing 2 nanomoles of [^13^C_3_]DG 36:2 (Larodan, Monroe, MI, USA) and 5 nanomoles [31H2]PE 16:0/18:1 (Avanti Polar Lipids, Alabaster, AL, USA). Two milliliters of methyl tert-butyl-ether was added to the sonicated pellets, followed by shaking at room temperature for 30 min (Thermo Fisher Multitube Vortexer, Waltham, MA, USA), and subsequent centrifugation at 4000× *g* for 30 min at room temperature. From the upper organic layer of these centrifuged samples, 1 mL aliquots were transferred to a deep-well microplates and dried via vacuum centrifugation (Eppendorf Vacfuge Plus, ThermoFisher Scientific, Waltham, MA, USA) and stored at 20 °C.

### 2.2. Lipidomics Analysis

To each dried sample, 200 μL infusion solvent was added. The infusion solvent consists of 2-propanol/methanol/chloroform (8:4:4 ratio), containing 5 mM ammonium chloride [22,24,25]. Lipids were characterized by flow infusion analysis (FIA) with electrospray ionization (ESI). FIA at 20 µL/minute was performed utilizing high-resolution (140,000 at 200 amu) data acquisition with an orbitrap mass spectrometer (Thermo Q Exactive, Waltham, MA, USA). The FIA included two 20-s scan epochs in positive electrospray ionization (PESI) and two 20-s scan epochs in negative electrospray ionization (NESI). In both cases, the first and second scan windows were 300 to 1000 amu and 999 to 2010 amu, respectively.

Between sample injections, the syringe and tubing were flushed with 1 mL of methanol followed by 1 mL of hexane/acetate/chloroform/water (3:2:1:0.1 ratio). FIA has the advantages of high sample throughput with a short analysis time and data acquisition, with a constant concentration of the lipid matrix.

Based on our infusion solvent, the predominant ions were [M+H]^+^, [M-H_2_O+H]^+^ or [M+NH_4_]^+^ in PESI, while they were [M-H]^−^ or [M+Cl]^−^ in NESI, where M is the exact mass of each lipid and +H is addition of a proton while -H is loss of a proton. For MS^2^ analyses, precursor ions were selected with a 0.4 amu window and collision energies of 15, 30 and 50 arbitrary units. Product ions were monitored with a resolution of 240,000 [28,29].

### 2.3. Data Reduction

Mass spectrometric data were imported into an Excel spreadsheet containing our in-house master lipidomics database of over 12,000 individual lipids from 193 different lipid families. The imported data included individual scanned masses and their associated peak intensities, which were then matched to lipids in the master database provided that the error was <2.0 ppm. For positive hits, the extracted mass and the associated peak intensity were imported into a new active spreadsheet if the peak intensity was >100,000 integrated counts (signal/noise > 3). For positive hits, the extracted mass and the associated peak intensity were imported into a new active spreadsheet if the peak intensity was >100,000 integrated counts (signal/noise > 3). Data are presented as a rank order with the most intense peak being assigned a value of 1.0. For the most intense peak, its relative levels (Relative level = endogenous lipid peak area/peak area of 2 nmoles [^13^C_3_]DG 36:2) are included in brackets in the Supplementary Tables.

Since there is no common “housekeeping” lipid for all the microbial strains we examined, to assess potential ion suppression, we calculated the ratio of the 2 internal standards in each extraction. For the PESI analyses, the [^31^H_2_]PE 34:1/[^13^C_3_]DG 36:2 ratio was 2.2 ± 10% while for the NESI analyses, the ratio was 1.6 ± 10%.

## 3. Results and Discussion

### 3.1. Consideration of Targeted vs. Non-Targeted Lipidomics Analyses

The ultimate goal of our research program is to establish a number of absolute quantitation assays for key lipid biomarkers of microbial infections, at the sub-threshold level, relative to clinical signs. This will be a laborious and expensive undertaking, since the analytical standards and stable isotope internal standards will generally require synthesis. Therefore, it is critical to identify and validate which lipid biomarkers represent a worthwhile investment of these resources. Non-targeted lipidomics, utilizing FIA-ESI, allows a broader analysis of the wide structural diversity of bacterial lipids that is not achieved with chromatographic methods. In addition, membrane adaptations that bacteria invoke with environmental changes can be monitored [30].

The utility of non-targeted analysis to identify potential bacterial lipid biomarkers has already been demonstrated, e.g., unique lipid biomarkers in the serum of cattle with paratuberculosis [26] and mycolic acid biomarkers useful for the characterization of *Gordonia* spp. in human sputum samples [31]. While a number of studies have focused on some oral bacterial lipid families, there is currently no comparative lipidomics studies of oral microbiota. This study represents the first step in this effort. Optimistically, unique lipid biomarkers associated with oral microbial dysbiosis can lead to potential diagnostic tests while increasing our understanding of the interactions between oral microbial species [6,7,9,10].

The diversity of microbial lipids is discussed next. Detailed information on each lipid is presented in the Supplementary Tables. This includes the lipid exact mass, monitored ions, and levels for each sample.

### 3.2. Modified Fatty Acyls: Aminoacyl Hydroxy-Fatty Acids (HFAs) in Gram-Negative Bacteria

Gly-HFA 16:0 (commendamide) was the predominant Gly-HFA family member across a number of Gram-negative oral bacteria investigated in this study. Commendamide was first reported for *Bacteroides* spp. isolated from GI microflora [32,33,34]. This is the first report of commendamide in oral microflora (Table 2), with MS^2^ analyses confirming the structure via generation of the product ions [Gly = 74.0248]^−^ and [Gly-CO-CH_2_ = 116.0353]^−^.

The roles of this bacterial endocannabinoid agonist in oral and GI function remain to be established.

### 3.3. Modified Fatty Acyls: Gly-Ser Lipids (Gly-Ser-FAHFA) in Gram-Negative Bacteria

Fatty acyls of hydroxy fatty acids (FAHFAs) have a hydroxy fatty acid (HFA) backbone and an acyl fatty acid substituent of the hydroxy group. FAHFAs include diverse lipid families with in-chain- and omega-hydroxy fatty acids [35,36,37,38,39]. FAHFAs with 5- and 9-HFA backbones are potent endogenous anti-inflammatory and anti-diabetic lipids [35,36,37]. ω-FAHFAs, also termed (O-acyl)-ω-hydroxy-fatty acids, act as surfactants in tear film [38], sperm and seminal fluid [40], and amniotic fluid [41]. While FAHFAs with a 3-HFA backbone are predominant in bacteria, FAHFAs with a 2-HFA backbone, also termed alpha-hydroxy fatty acids (AAHFAs) possessing acyl substituents of propionic and butyric acids, have recently been described in gut microbiota [39]. In the case of Gly-Ser-FAHFAs, they have a 3-HFA backbone with Gly-Ser-FAHFA 32:0 (Flavolipin, Lipid654; PubChem CID 53787314) the dominant member of this lipid family (Table 3).

The Gly-Ser lipids have been extensively studied in the laboratory of Dr. F. C. Nichols, including the synthesis of analytical standards and stable isotope internal standards for absolute quantitation [42]. These dipeptide lipids have been recovered from periodontitis samples [43,44,45,46], arteries [44] and human serum [43]. In these clinical cases, the Gly-Ser lipids are virulence factors present in outer membrane vesicles [47,48,49] and are TLR2 ligands [45]. Lower serum levels of the predominant family member, Gly-Ser-FAHFA 32:0, have been monitored in both multiple sclerosis and Alzheimer’s patients [43]. These researchers have suggested that this results in altered brain microglial function [50], a cellular pathway in neurodegeneration. In addition to Gly-Ser FAHFAs in some Gram-negative bacteria, phosphor-glycerol serine-glycine lipo-dipeptides (Gly-Ser-FAHFA-P-DG) have also recently been discovered [46]. In our study we only monitored these complex lipids in *P. gingivalis* and *F. nucleatum* (Table 3).

As described by previous studies [45,46,47,51], Gly-Ser lipids were monitored in Gram-negative Bacteroidetes bacteria in this study. The rank order of Gly-Ser-FAHFA lipids in *P. gingivalis* was 32:0 > 31:0 > 30:0 > 33:0 > 34:0 > 28:0 (Table 3 and Supplementary Tables). Gly-Ser-FAHFA 32:0, the predominant Gly-Ser lipid [52], is found in the outer membranes of a number of Gram-negative bacteria [53].

**Figure 1:**
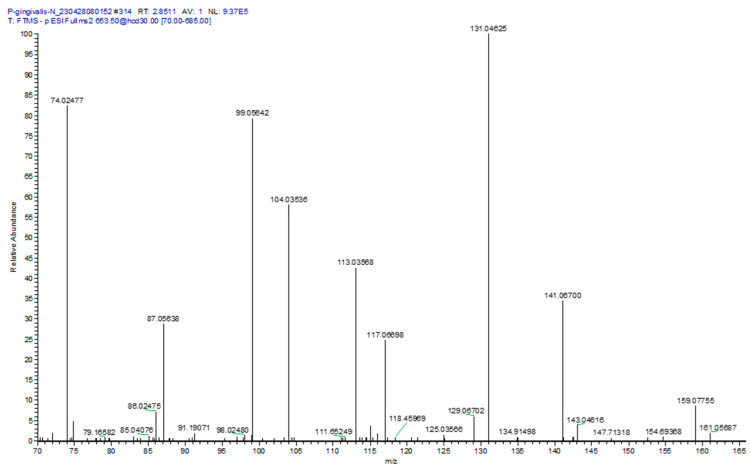
MS^2^ product ion spectrum for Gly-Ser-FAHFA 32:0, [653.5]^−^ extracted from *P. gingivalis.* Specific product ions included glycine (74.02477, 0.68 ppm); serine (104.03536, 0.38 ppm); serine minus H_2_O (86.02475, 0.12 ppm); Gly-Ser (161.05687, 0.56 ppm); Gly-Ser minus H_2_O, (143.04616, 0.42 ppm); and Gly-Ser minus CO_2_, (117.06698, 0.26 ppm). The only fatty acid in the product ion spectrum was FA 15:0 (241.21729, 0.30 ppm), supporting the published structure for Gly-Ser-FAHFA 32:0 as Gly-Ser-FAHFA(17:0/O-15:0).

The structures of the Gly-Ser lipids in our study were validated by MS^2^, which generated the product ions for MS^2^ of Gly-Ser-FAHFA 32:0: Glycine (74.02477; 0.27 ppm), Serine (104.03537, 0.676 ppm), Gly-Ser (161.05687/143.04616), FA 15:0 (241.21729, 0.041ppm), M—FA 15:0 (411.28630, 0.85 ppm), and HFA 17:0 (285.2435, 0.25 ppm) (Figure 1). In the case of the predominant Gly-Ser-FAHFA 32:0, the acyl substituent fatty acid product was clearly FA 15:0 and the HFA 17:0, supporting the structure 15:0-17:0(OH)-Gly-Ser, with the fatty acylation at hydroxyl function of HFA 17:0 (PubChem CID 53787314).

At the masses of Gly-Ser-FAHFA 30:0 to 33:0, the product ions of asparagine and glutamine were also monitored (Figure 1). These presumably are products of Gln-hydroxy-FAHFA 30:0 and Asn-hydroxy-FAHFA 31:0 for the Gly-Ser-FAHFA 30:0 mass and Gln-hydroxy-FAHFA 32:0 and Asn-hydroxy-FAHFA 33:0 for the Gly-Ser-FAHFA 32:0 mass. Validation of acylation with a HFA, rather than a FA, was the product ion for M—HFA 16:0 (381.27578, 0.19 ppm). The loss of this HFA 16:0 acyl substituent tentatively identifies Gln-hydroxy-FAHFA 30:0 as Gln-FAHFA 14:0/O-16(OH), Asn-hydroxy-FAHFA 31:0 as Asn-FAHFA 15:0/O-16(OH), Gln-hydroxy-FAHFA 32:0 as Gln-FAHFA 16:0/O-16(OH), and Asn-hydroxy-FAHFA 33:0 as Asn-FAHFA 17:0/O-16(OH). These data support previous observations of Gln- hydroxy-FAHFA in gut microbes [33].

Gln- and Orn-FAHFAs have been reported previously in Gram-negative bacteria [33]. In our study we only monitored Gln-FAHFAs in *P. gingivalis*, representing a unique lipid biomarker for this microbe. The product ions (Figure 1) for both asparagine- and glutamine-FAHFAs were monitored in the MS^2^ analysis of Gly-Ser-FAHFA 32:0 (653.5). The specific ions included Asn (131.04625, 0.22 ppm); Asn minus H_2_O (113.03568, 0.18 ppm), Asn minus CO_2_ (87.05638, 0.080 ppm), and Gln minus H_2_O and CO (99.05642, 0.30 ppm). The only hydroxy fatty acids (HFA) detected in the product ion spectrum were for HFA 17:0 minus H_2_O (267.23274, 0.82 ppm) and HFA 18:0 minus H_2_O (281.24833, 0.99 ppm). The tentative structures at this mass of 653.5 could be Asn-hydroxy-FAHFA 33:0 (Asn-FAHFA(16:0/O-17:0(OH) or Asn-FAHFA 15:0/O-18:0(OH)) and Gln-hydroxy-FAHFA 32:0 (Gln-FAHFA(14:0/O-18:0(OH) or Gln-FAHFA(15:0/O-17(OH)).

Orn-FAHFAs were also monitored in *P. ginivalis*, *T. denticola*, and *A. muciniphilia* (Supplementary Tables). The MS^2^ product ions [133.09715/115.08659]^+^ validated the amino acid component as ornithine.

**Figure 2:**
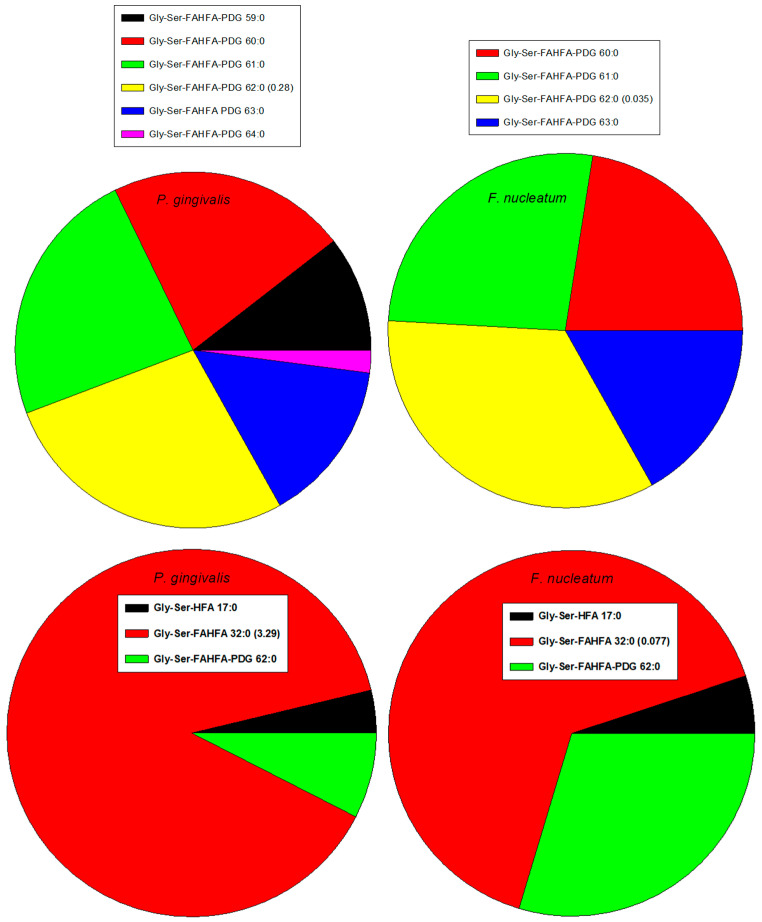
Rank orders of Gly-Ser lipids in Gram-negative *P. gingivalis* and *F. nucleatum*. Relative levels of the dominant family member relative to the internal standard, 2 nmoles of [^13^C_3_]DG 36:2. HFA, hyfroxy fatty acid; PDG, phosphor-diacyl-glycerol.

Gly-Ser-FAHFA 32:0 and Gly-Ser-FAHFA-P-DG 62:0 have been monitored in several Gram-negative classes of the phylum Bacteroidota, including Bacteroida (*P. gingivalis*, *Porphyromonas endodontalis*, *Prevotella intermedia*, *Tannerella forsythia*, *Bacteroides fragilis*. *Bacteroides ovatus*, *Bacteroides vulgatus*, *Bacteroides thetaiotaomicron*) and Flavobacteriia (*Capnocytophaga sputigena*, *Capnocytophaga gingivalis*, *Capnocytophaga ochracea*) [39,47,54,55]. In contrast, these Gly-Ser lipids were not detected in Gram-negative classes of the phylum *Proteobactia,* including *Gammaproteobacteria* (*Aggregatibacter actinomycetemcomitans*), the phylum *Fusobacteriota,* including *Fusobacteriia* (*F. nucleatum*), and the phylum *Spirochaetota,* including *Treponema denticola* [47]. We also monitored Gly-Ser-FAHFA 32:0 in Bacteroida (*P. gingivalis*, *Prevotella brevis*) but, in contrast to previous reports, we also monitored this lipid in *T. denticola* (0.43% of *P. gingivalis* levels) and *F. nucleatum* (2.34% of *P. gingivalis* levels) (Figure 2). This may relate to life cycle differences or a difference in assay sensitivity. Gly-Ser-FAHFA-P-DG 62:0 was only monitored in *P. gingivalis* and *F. nucleatum* (12.5% of *P. gingivalis* levels). We did not monitor these Gly-Ser lipids in the Gram-negative phylum Verrucomicrobiota, class Verrucomicrobiae (*Akkermansia muciniphila*) or Bacillota, class Clostridia (*Veillonella parvula*).

We monitored Gly-FAHFA 32:0 only in *P. gingivalis* and Gly-Ser-HFA 17:0 in *P. gingivalis* and *F. nucleatum* (0.41% of *P. gingivalis* levels). These metabolites/precursors of Gly-Ser-FAHFA 32:0 and Gly-Ser-FAHFA-P-DG 62:0 have been previously monitored in Bacteroidota [47,54].

Recently, a new family of phosphor-glycerol Gly-Ser-FAHFAs in *P. gingivalis* has been reported, which are also TLR2 ligands [46]. We also monitored these lipids in *P. gingivalis* and in *F. nucleatum* (Table 3) with the dominant family member Gly-Ser-FAHFA-P-DG (62:0). Lipid identities of Gly-Ser-FAHFA-P-DGs were validated with the MS^2^ product ions listed in Table 4.

Gly-Ser-hydroxy-fatty acids (Gly-Ser-HFA), which may be metabolites and/or precursors of Gly-Ser FAHFAs, have also been reported in Gram-negative bacteria [48,52]. We only detected these lipids in *P. gingivalis* with Gly-Ser-HFA 17:0 being the dominant family member. Structural validation was obtained with the product ions for glycine, serine, Gly-Ser, and HFA 17:0, all with <1 ppm mass error. Of significant relevance to these findings are the early astute observations of significant levels of 3-hydroxy fatty acid 17:0 in periodontitis [56], again, a possible metabolite and/or precursor of Gly-Ser FAHFAs, since the HFA in these lipids is 3-HFA 17:0.

### 3.4. Glycerolipids (GL) and Modified-GLs

The monoacylglycerols monitored were mainly 16:0, 18:0, and 18:1, with the highest levels in *C. albicans.* In contrast, diacylglycerol (DG) levels were highest in *F. nucleatum*, which also expressed the most diverse array of DGs. Alanyl-DGs were monitored in all Gram-positive bacteria except for *S. oralis* and *S. gordinii*. TGs were highest in *S. acidominus*, *P. brevis* and *C. albicans* (Supplementary Tables).

**Figure 3:**
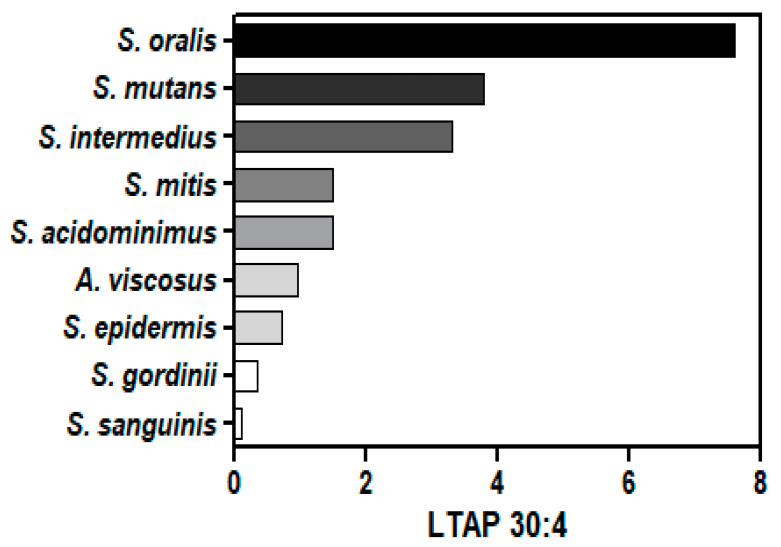
Relative levels of LTAP 30:4 (lipoteichoic acid precursor) in Gram-positive bacteria. The internal standard was 2 nmoles of [^13^C_3_]DG 36:2.

The unique modified glycerolipids of Gram-positive bacteria are dihexosyl DGs (DHDGs) serving as precursors of membrane lipoteichoic acids (LTA) [22,57,58,59,60,61]. We monitored these unique glycolipids in all Gram-positive bacteria, as well as lipoteichoic acid precursor (LTAP) 30:4 (Figure 3; Supplementary Tables). LTAP involves the addition of glycerol phosphate to DHDG resulting in DHDG-GroP (LTAP). MS^2^ experiments validated the LTAP identities with product ions for GroP (171.0059/152.9953)^−^ and HexosylGroP-H_2_O (315.0481).

Trihexosyl diacylglycerols (THDG), which serve as lipid anchors of cell surface LTAs, are involved in immunomodulation and as possible precursors of LTAs [62,63,64,65]. These lipids have been found only in *Romboutsia* spp., *C. difficile*, and *Lactobacillus casei* [45,62,63,64,65,66]. We monitored, for the first time, these lipid anchors in all of the Gram-positive bacteria we studied except for *S. acidominimus* (Supplementary Tables). The predominant family member was trihexosyl-DG 32:0. MS^2^ experiments validated the trihexosyl-DG identities with product ions for trihexosyl-glycerol [577.19346/559.1829]^−^ and Hexose [179.0561]^−^.

### 3.5. Glycerphospholipids (GPLs)

In general, while phosphatidylcholines (PCs) were detected across Gram-negative and Gram-positive bacteria, phosphatidylethanolamines were more prevalent in Gram-negative bacteria. Plasmalogens, phosphatidylethanolamines, and phosphatidic acids were only monitored at very low levels. Lysophosphatidic acid 16:0 was uniquely monitored at high levels in *C. albicans* (Supplementary Tables).

The dominant phosphatidylcholine (PC) in oral microflora was PC 34:1 with the highest levels in *C. albicans*, *S. acididominimus*, and *C. albicans* (Table 5). These microflora, along with *P. vulgaris* (high levels of PC 30:1) and *N. lyticus* (high levels of PC 30:0), have lysophosphocholine levels that are fractions of the PC levels (Table 5), similar to observations in eukaryotes. In sharp contrast to eukaryotes, a larger number of oral microflora have atypical LPC levels that are multiples of the endogenous PC levels (Table 5). These data suggest that LPCs may play unique roles in the membranes of these microflora and are not just degradation products or precursors of PCs.

Another unique feature of bacterial PCs, compared to eukaryotes, is the absence of PCs with polyunsaturated fatty acids. This more limited variation in PC lipids in bacteria may contribute to a more rigid cell membrane and altered lipid raft function.

Structural identities of PCs and LPCs were validated in PESI with the product ion for phosphocholine (184.0738). This is important, since odd carbon phosphatidylethanolamines would be monitored at PC masses in PESI (e.g., PC 32:0 = PE 35:0 = 733.5622).

**Figure 4:**
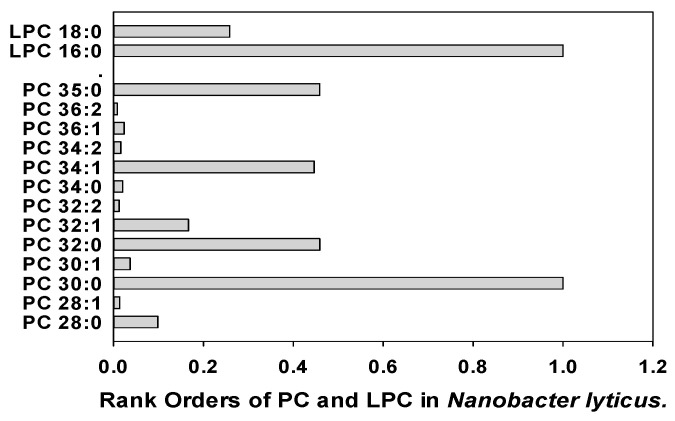
Rank orders of phosphatidylcholines (PC) and lysophosphatidylcholines (LPC) in *Nanobacter lyticus*.

Nanobacter lyticus (TM7x) was included in these analyses since this ultrasmall bacterium (200 to 300 nm), which possesses a Gram-positive cell envelope, survives as an epibiont on the surfaces of larger oral bacteria [67,68] and is present in human saliva [69,70]. Our data are the first lipidomics characterization of this important oral bacteria and demonstrate the major lipid family produced by the more compact genome of this bacteria is glycerol-phosphocholines (Figure 4; Supplementary Tables).

Phosphatidylglycerols (PGs) were monitored at low levels in all microflorae with no one family member being dominant (Supplementary Tables). In all cases, the MS^2^ products were [DG-H_2_O]^+^, indicating that the lipids were PGs and not the isobars acyl-lyso-PG, also termed semi-lysobisphosphatidic acid (SLBPA) and bis(monoacylglycerol)phosphate (BMP) in the literature.

### 3.6. Sphingolipids: Ceramides

While ceramides and GroP-ceramides were more predominant in Gram-negative bacteria and *C. albicans*, deoxy-ceramides were monitored across oral microflora (Supplementary Tables). High levels of galactosyl dihydroceramides were monitored in *C. albicans*.

**Figure 5:**
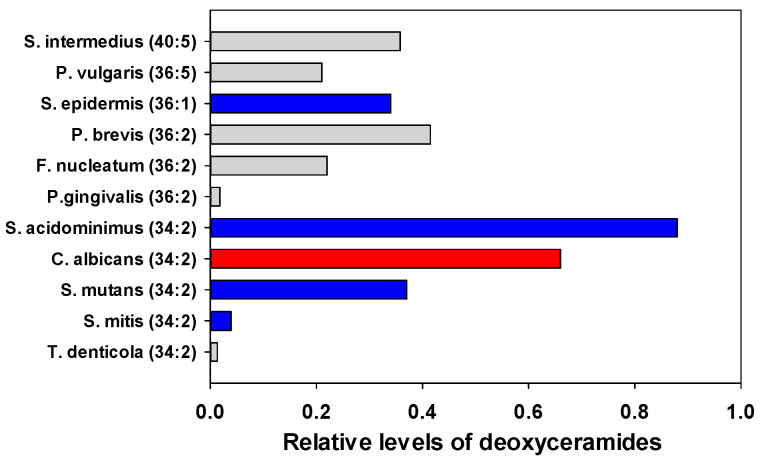
Relative levels of the most abundant deoxy-ceramides in oral microflora. The internal standard was 2 nmoles of [^13^C_3_]DG 36:2. Gray (Gram negative), Blue (Gram positive), Red (fungi).

Deoxy-ceramide sphingolipids lack the 1-hydroxy group of the sphingolipid headgroup. serine palmitoyl transferase is promiscuous and can utilize alanine rather than serine in the condensation reaction with a fatty acyl-CoA to generate deoxy-ceramides rather than a ceramide. Deoxy-ceramide 34:2 (Cer 34:2;O) was the dominant family member with highest levels in *C. albicans*, *S. acidominimus*, and *S. mutans* (Figure 5). Lipid identities were confirmed by the dehydrated deoxy-sphingosine bases as MS^2^ products in PESI.

### 3.7. Sphingolipids: Sphingomyelins

**Figure 6:**
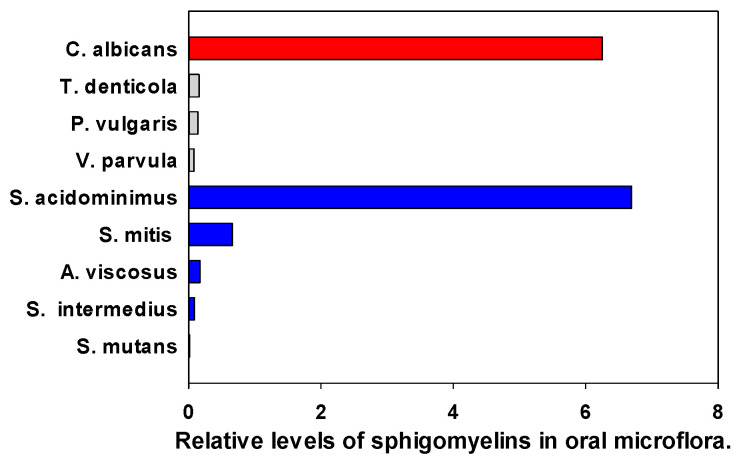
Relative levels of the sphingomyelin 34:1;O2 (SM d18:1/16:0) in oral microflora. The internal standard was 2 nmoles of [^13^C_3_]DG 36:2. Gray (Gram—negative), Blue (Gram—positive), Red (fungi).

Sphingomyelin levels were found to be low except for *C. albicans* and *S. acidominimus* (Figure 6). In all cases, the MS^2^ product was (Phosphocholine = 184.0733)^+^, indicating that the lipids were SMs and not the isobars ceramide-phospho-ethanolamines (PE-Cer) or ceramide aminoethyl-phosphonates (CAEP).

### 3.8. Sphingolipids: Phosphorylated Ceramides

PE-ceramides are lipid biomarkers of several Gram-negative genera, including *Bacterioides*, *Porphyromonas*, *Prevotella*, *Tanneralla*, and *Parabacteroides* [66,71,72,73,74] and are constituent lipids in insects [75]. PE-ceramides have been monitored in human gingival tissues, blood, vascular tissues, and brain [71,76]. Consistent with this, we only monitored PE-ceramides in extracts of the *Bacterioides P. gingivalis*, *P. brevis*, and *P. vulgaris*. PE-Cer 35:0;O3, was tentatively identified as PE-Cer d18:0/h17:0 based on the MS^2^ product ions (PE = 140.0118; 0.58 ppm)^−^ and (HFA 17:0 = 285.2435; 0.073 ppm)^−^. Cer d18:0/h17:0, also termed pecipamide (Lipid Maps, LMSP02020019), has been monitored in the fungus *Polyporus picipes* [77]. This is the first report of a PE-modification of this lipid, which we detected in *P. gingivalis* (Supplementary Tables).

PI-ceramides (IPC) are also present in *Bacterioides* spp. [73]. However, we only monitored these GPL-modified ceramides and their mannosyl derivatives in *C. albicans* (Supplementary Tables). These data are consistent with previous evaluations of IPCs and mannosyl IPCs in fungi [78,79,80,81].

### 3.9. Sphingolipids: Ceramide Sulfonates

Ceramide sulfonates are sulfonolipids of *Bacteroida* spp. and *Flavobacteria* spp. The sphingosine base is replaced by capnine, a product of cysteic acid and fatty acyl-CoA [51,80]. These lipids, which have also been termed sulfobacins, like Sulfobacin A (Pubchem CID 10438855), have been monitored in Gram-negative GI *Bacteroidetes*, *Alstipes* and *Odoribacter* spp. [82,83].

In agreement with *Bacteroida* spp. generating sulfonolipids, we monitored Cer 33:2;O2 Cer 34:2;O2, Cer 35:2;O2, and Cer 35:2;O2 sulfonates in *P. gingivalis* and Cer 36:2;O2, Cer 37:2;O2, and Cer 38:2;O2 sulfonates, as well as Cer 35:1;O3 sulfonate in *P. brevis* (Supplementary Tables). Additionally, Cer 33:1;O3, 34:1;O3 (Sulfobacin SL3; LipidMaps LMSP00000021), and Cer 35:1;O3 sulfonates were monitored in *C. albicans* (Supplementary Tables). This is the first report of this lipid family in a fungus.

The roles of these unique highly charged lipids in oral and gut microbes remain to be established. However, the location of sulfonolipids in the cell envelope of *Cytophags* spp. suggests that they may contribute to membrane charge in some Gram-negative bacteria [84].

### 3.10. Glycopeptidolipids (GPL)

GPLs have been reported for a number of non-tuberculosis-causing *Mycobacteria*. These large molecular weight GPLs have a tripeptide-amino-alcohol core (Phe-Thr-Ala-Alaninol) with a 3-hydroxy or a 3-methoxy C26-C33 fatty acyl chain N-linked to the Phe.

Glycosylation includes 6-deoxytalose bonded to Thr and rhamnose boned to alaninol [85]. We monitored GPLs in Gram-positive *A. viscosus* with the dominant GPL being h36:1(DiAc-dTal)-Phe-Thr-Ala-Alaninol-TriMe-Rham [1345.9347]^+^.

GPLs are thought to be involved in virulence, biofilm formation, and sliding behavior [85]. In this regard, GPLs may contribute to secretions involved in the sliding behavior of *A. viscosus* [86].

### 3.11. Mutanamides: Lipopeptides

**Figure 7:**
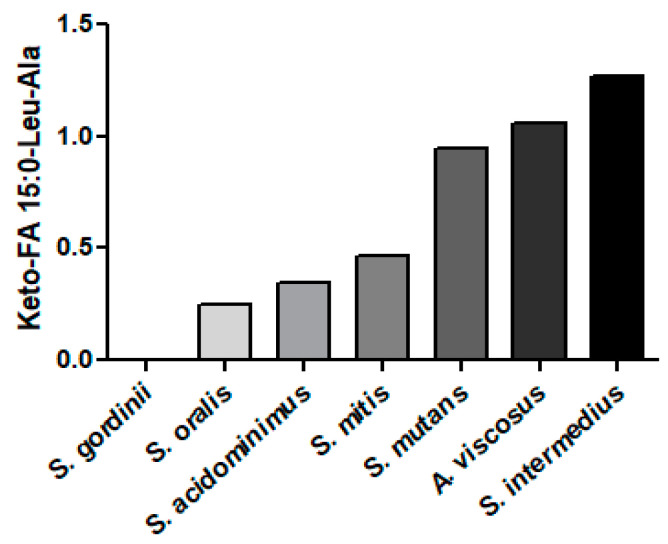
Relative levels of mutanamide (keto-FA 15:0-Leu-Ala) in Gram-positive bacteria. The internal standard was 2 nmoles of [^13^C_3_]DG 36:2.

The Gram-positive bacterium *S. mutans* acylates dipeptide products of non-ribosomal origin. Examples of this are the mutanamides, where Leu-Ala is acylated with keto fatty acids of various carbon lengths [87]. In our analyses, we detected a number of mutanamides in Gram-positive but not Gram-negative bacteria or in *C. albicans* (Figure 7). Highest levels were monitored in *S. intermedius*, *A. viscosus*, and *S mutans*. The extent of the biological activity of these novel lipopeptides remains to be explored, but we do know that they inhibit fungal hyphal formation [87].

Mutanamides consist of a keto fatty acid backbone of various carbon lengths which are N-acylated with the dipeptide Leu-Ala [[87], LMFA08020297]. The dominant mutanamide that we monitored in Gram-positive bacteria was ketoFA 15:0-Leu-Ala. The MS^2^ products of these lipopeptides were alanine [90.05495]^+^ and leucine [132.1019; 86.0964]^+^.

### 3.12. Betaine Lipids: Monoacylglyceryl-Carboxyhydroxymethylcholine (MGCC)

**Figure 8:**
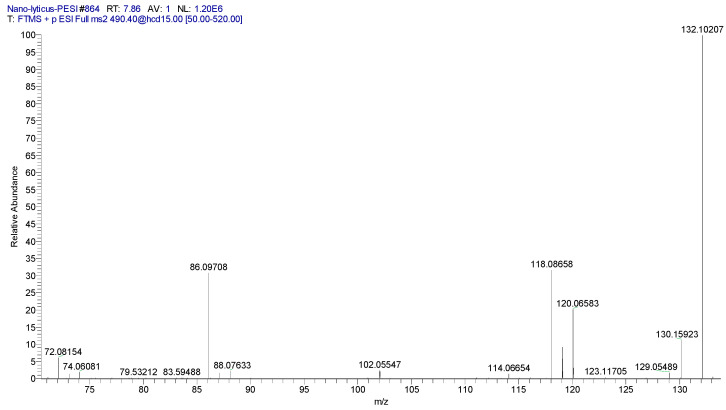
MS^2^ product ion spectrum for MGCC 16:0 (490.4)^+^. Specific product ions for the betaine headgroup included (C_4_H_10_N = 72.0813; 3.0 ppm)^+^, (C_5_H_12_N = 86.0969; 1.2 ppm)^+^, (C_5_H_12_NO = 102.0918; 0.38 ppm)^+^, and (C_6_H_14_NO_2_ = 132.1024; 2.9 ppm)^+^.

MGCCs (also termed lyso-DGCC) are polar lipids that can substitute for phosphorylcholines in membranes. These lipids have been monitored in microalgae [88,89,90], copepods [89], and corals [91]. Our data are the first to detect these lipids in a bacterium, specifically *N. lyticus* (Table 6). The glycerophospholipid profile of N. lyticus is limited, with PCs dominating. DGCCs may function as a lipid reservoir that can substitute for PCs during cellular stresses. MGCC identities were validated by MS^2^ (Figure 8).

### 3.13. Unique Fungal Lipid Biomarkers

In our study, four lipids distinguished *C. albicans* from all monitored bacteria. These were ergosterol, inositol phospho-ceramides, sulfo-phosphatidylglycerols, and lysophosphatidic acids.

Ergosterol was only monitored, as expected, in *C. albicans* but not bacteria (Supplementary Tables). The MS^2^ product ions (C_19_H_24_ = 253.1951; 0.32 ppm)^+^ and (C_23_H_32_ = 309.2577; 0.23 ppm) validated the identity of ergosterol.

The fungal inositol phosphoceramides (IPC) again were monitored in *C. albicans* but not bacteria (Supplementary Tables). IPC 38:0;O was the dominant member of this lipid family.

Unexpectedly, sulfo-phosphatidylglycerols (Sulfo-PGs) were detected in *C. albicans* but not bacteria (Supplementary Tables). The dominant family member was sulfo-PG 38:0. Previously, these sulfated lipids have only been reported for archaebacteria [91]. The prevalence of sulfo-PGs in other fungi and their functions remain to be explored. While the sufonolipid sulfobacins (N-acylated capnine) have also been reported for bacteria [83,84] and fungi (see Section 3.9), this is the first report of sulfo-PGs in a fungus.

While lysophosphatidic acids (LPA) are also present in bacteria, the only robust LPA levels monitored in this study were in *C. albicans* (Supplementary Tables).

## 4. Summary

Microbial lipids have a broader range of structural diversity and complexity, compared to the mammalian lipidome. Species-specific lipid modifications (e.g., glycosylation, incorporation of amino acids and peptides) provide the potential to identify lipid biomarkers for bacteria and fungi. Lipid biomarkers can serve as research tools in the study of biofilm production and functions, pathogenic lipids, cell wall/envelope molecular adaptation, and detection of sub-clinical microbial infections.

The extensive diversity of lipid modifications was clearly demonstrated in our study. The lipid scaffolds of glycerol (glycerolipids, glycoglycerolipids, glycerophospholipids, and glycerophospholipid modified ceramides), sphingosine (ceramides, sphingomyelins), and FAHFAs (Gly-, Gly-Ser-, Orn-, Asn-, Gln-FAHFA) were all found to possess a significantly greater molecular diversity than in mammals, making them valuable biomarkers. A summary of the specific lipid findings is presented in Table 7.

## 5. Limitations

Biological Limitations: Bacteria and fungi have complex life cycles and their lipidomes will vary with those cycles. We only take a snapshot of one point in time with our commercial samples. However, these analyses will allow us to define major unique lipid families in each bacterial strain. Bacterial expression in their hosts will be complex but a range of members of each unique lipid family has the possibility of being monitored and can be optimized by longitudinal sample collections [9]. In addition, while we obtained detailed lipidomics data for 17 oral bacteria and 1 oral fungus, the oral cavity has over 600 bacterial species and 100 fungal species. This necessitates efforts to continue to expand this first lipidomics database for oral microbes. Such a database is essential for studies of bacterial adaptation to environmental changes, including the development of resistance to antibiotics.

Technical Limitations: Our HR-MS analytical platform (≤2 ppm mass error), which utilizes both PESI and NESI, significantly reduces the risk of lipid misassignments. However, there are a number of lipid structural isobars that require MS^2^ and/or TLC evaluations for full structural validation. Over the last 10 years, our Metabolomics Unit has built a database of a number of these specific issues and optimal technical solutions. Specific issues include our inability to distinguish between: (i) a cyclopropyl group and a double bond in a fatty acid chain, and (ii) an added methyl group vs. addition of a CH_2_ in a fatty acid. Again, MS^2^ and/or TLC evaluations will be our first strategies with lipids of high interest. NMR may be considered if required, but this involves significant scale-up and purification methods due to the lower sensitivity of NMR.

## Figures and Tables

**Table 1:** List of the commercial microflora utilized in this lipidomics study.

Microbe	ATCC #	Gram	Class
*Streptococcus oralis*	9811	Positive	*Bacilli*
*Streptococcus intermedius*	27335	Positive	*Bacilli*
*Streptococcus mitis*	49456	Positive	*Bacilli*
*Streptococcus sanguinis*	10556	Positive	*Bacilli*
*Streptococcus gordonii*	33399	Positive	*Bacilli*
*Streptococcus mutans*	35668	Positive	*Bacilli*
*Staphylococcus epidermis*	14990	Positive	*Bacilli*
*Streptococcus acidominimus*	51726	Positive	*Bacilli*
*Actinomyces viscosus*	43146	Positive	*Actinomycetia*
*Nanosynbacter lyticus*	TSD 290	Positive	*Saccharimonadia*
*Porphyromonas gingivalis*	33277	Negative	*Bacteroidia*
*Prevotella brevis*	19188	Negative	*Bacteroidia*
*Fusobacterium nucleatum*	10953	Negative	*Fusobacter*
*Veillonella parvula*	10790	Negative	*Clostridia*
*Treponema denticola*	35405	Negative	*Spirochaetia*
*Proteus vulgaris*	8427	Negative	*Gammaproteobacteria*
*Alkermansia muciniphila*	BAA 835	Negative	*Verrucomicrobiae*
*Candida albicans*	24433	Fungus	*Sachcharomycetes*

# Catalog number.

**Table 2:** Glycine hydroxy fatty acids (HFA), including commendamide (Gly-3-HFA 16:0), found in only 3 of the 8 Gram-negative bacteria. Data are presented as Rank orders with the relative levels of the most abundant family member to 2 nmoles of [^13^C_3_]DG 36:2 in parentheses. Blanks represent Not Detected.

Gly-Hydroxy-FA	*P. gingivalis*	*F. nucleatum*	*T. denticola*
Gly-HFA 15:0		0.017	0.022
Gly-HFA 16:0	1.0 (0.042)	0.825	1.0 (0.040)
Gly-HFA 17:0			0.036
Gly-HFA 18:0	0.109	0.115	0.141
Gly-HFA 19:0	0.047	1.0 (0.036)	0.119
Gly-HFA 20:0	0.047	0.038	0.051

**Table 3:** Gly-Ser-FAHFAs and the Gly-Ser-FAHFA-phospho-diacyl-glycerols (Gly-Ser-FAHFA-P-DG). Data are presented as Rank orders with the relative levels of the most abundant family member to 2 nmoles of [^13^C_3_]DG 36:2 in parentheses. Blanks indicate not detected.

Gly-Ser-FAHFA	*P. gingivalis*	*F. nucleatum*	*T. denticola*
Gly-Ser FAHFA 27:0			0.0239
Gly-Ser FAHFA 30:0	0.0954		
Gly-Ser FAHFA 31:0	0.3595		0.1983
Gly-Ser FAHFA 32:0	1.0 (3.29)	1.0 (0.077)	1.0 (0.014)
Gly-Ser FAHFA 33:0	0.0377		
**Gly-Ser-FAHFA-P-DG**			
Gly-Ser-FAHFA-P-DG 59:0	0.3845		
Gly-Ser-FAHFA-P-DG 60:0	0.7934	0.6614	
Gly-Ser-FAHFA-P-DG 61:0	0.8599	0.7809	
Gly-Ser-FAHFA-P-DG 62:0	1.0 (0.28)	1.0 (0.035)	
Gly-Ser-FAHFA-P-DG 63:0	0.5418	0.4949	
Gly-Ser-FAHFA-P-DG 64:0	0.0753		

**Table 4:** MS^2^ validation of Gly-Ser-FAHFA-phospho-diacyl-glycerols (P-DG).

Lipid * ([M-H]^−^)	Product Ions ([M-H]^−^, ppm)
Gly-Ser-FAHFA-P-DG 59:0 (1213.8952)	PA 28:0 (591.40314, 0.017 ppm) PA 28:0 minus FA 13:0 (395.22038, 0.094 ppm) FA 13:0 (213.18612, 0.54 ppm)
Gly-Ser-FAHFA-P-DG 60:0 (1227.9109)	PA 28:0 (591.40290, 0.39 ppm) PA 28:0 minus FA 15:0 (367.18918, 0.14 ppm) FA 15:0 (241.217238, 0.27)
Gly-Ser-FAHFA-P-DG 61:0 (1241.9265)	PA 29:0 minus FA 13:0 (409.23602, 0.15 ppm) FA 13:0 (213.18604, 0.64)
Gly-Ser-FAHFA-P-DG 62:0 (1255.9422)	PA 30:0 (619.43440, 0.052 ppm) PA 30:0 minus FA 15:0 (377.20984, 0.032 ppm) FA 15:0 (241.21729, 0.30 ppm)
Gly-Ser-FAHFA-P-DG 63:0 (1269.9578)	PA 31:0 (633.44928, 1.26 ppm) PA 31:0 minus FA 15:0 (409.23596, 0.29 ppm) FA 15:0 (241.21736, 0.23 ppm)
Gly-Ser-FAHFA-P-DG 64:0 (1283.9735)	Signal too weak

* All lipids had a glycerol phosphate ion (152.9957, 0.31 to 0.34 ppm). FA, fatty acid; PA, phosphatidic acid; ppm, parts-per-million mass error.

**Table 5:** Relative levels of the most abundant PC family members to 2 nmoles of [^13^C_3_]DG 36:2. Relative LPC levels and the ratios of these to the PCs are presented. Blanks represent Not Detected.

Microbe	PC 30:0	PC 34:1	PC 30:1	PC 32:0	PC 34:0	PC 36:3	LPC 16:0	LPC 18:0	LPC 16:1	LPC/PC
*S.sanguins*		0.300						0.31		1.03
*S. mutans*		0.047						0.33		7.02
*S. acidominus*		7.80					0.18			0.023
*A. viscosus*		9.69						0.95		0.098
*P. gingivalis*		0.610					1.13			1.85
*F. nucleatum*		0.180					0.86			4.78
*T. denticola*		0.710					1.28			1.80
*C. albicans*		10.14							2.15	0.21
*S. epidermis*					0.27				0.65	2.38
*S. intermedius*					2.58			20.37		7.89
*P. vulgaris*			4.59				2.44			0.53
*P. brevis*			1.74				10.64			6.12
*V. parvula*			2.66				10.72			4.03
*S. oralis*	1.03						22.83			22.10
*N. lyticus*	9.10						0.339			0.037
*A.muciniphilia*						0.014	0.22			15.71
*S. mitis*				2.66				11.02		4.14
*S. gordinii*				1.12			22.72			20.29

**Table 6:** Relative levels of MGCCs to 2 nmoles of [^13^C_3_]DG 36:2 in *N. lyticus*. Relative LPC levels and the ratios of these to the PCs are presented.

MGCC	Exact Mass	(M+H)^+^	*N. lyticus*
MGCC 16:0	489.3665	490.3738	1.0 (0.12)
MGCC 18:0	517.3978	518.4051	0.417
MGCC 18:1	515.3821	516.3894	0.082
MGCC 20:1	543.4134	544.4207	0.247
MGCC 22:2	569.4291	570.4364	0.520
MGCC 24:1	599.4761	600.4833	0.223

**Table 7:** A summary of the presence or absence of specific lipid families in the microbes of this study. Since we set a rigorous threshold for the peak intensity for individual lipids, this summary does not include potential lipids present at lower concentrations. Green = present; Red = not detected.

	Fatty Acyle			Glycerolipids				Glycerphospholipids		Sphingolipids			Other Lipids		
	Gly-HFA	Gly-Ser-FAHFA	Gly-Ser-FAHFA-P-DAG	MG	DG	DHDG	THDG	Glycerophospho-cholines	Phosphatidyl-glycerols	Ceramides	PE-Cermides	Ceramide Sulfonates	Glycopeptidolipids	Mutanamides	Betaine Lipids
**Gram-Positive Commensal**															
*S. oralis*															
*S. intermedius*															
*S.mitis*															
*S.sanguinis*															
*S. gordonii*															
**Gram-Positive Opportunistic**															
*S. mutans*															
*S. epidermis*															
*S. acidominimus*															
*A. viscosus*															
*N. lyticus*															
**Gram-Negative Opportunistic**															
*P. gingivalis*															
*P. brevis*															
*P. vulgaris*															
*F. nucleatum*															
*V. parvula*															
*T. denticola*															
*A. muciniphila*															
**Fungus**															
*C. albicans*															

Green = Present; Red = Absent; Black = Not Monitored.

## Data Availability

All data is included in the manuscript and Supplementary Materials.

## Supplementary Materials

The following supporting information can be downloaded at: https://www.mdpi.com/article/10.3390/metabo14040240/s1, Excel spreadsheet of all lipid data.

## References

[1] Tuominen H., Rautava J. (2021). Oral Microbiota and Cancer Development. Pathobiology.

[2] Colombo A.V., da Silva C.M., Haffajee A., Colombo A.P. (2007). Identification of intracellular oral species within human crevicular epithelial cells from subjects with chronic periodontitis by fluorescence in situ hybridization. J. Periodontal Res..

[3] Teles F.R., Teles R.P., Uzel N.G., Song X.Q., Torresyap G., Socransky S.S., Haffajee A.D. (2012). Early microbial succession in redeveloping dental biofilms in periodontal health and disease. J. Periodontal Res..

[4] Herrero E.R., Slomka V., Bernaerts K., Boon N., Hernandez-Sanabria E., Passoni B.B., Quirynen M., Teughels W. (2016). Antimicrobial effects of commensal oral species are regulated by environmental factors. J. Dent..

[5] Borgnakke W.S., Ylöstalo P.V., Taylor G.W., Genco R.J. (2013). Effect of periodontal disease on diabetes: Systematic review of epidemiologic observational evidence. J. Clin. Periodontol..

[6] Dong J., Liu L., Chen L., Xiang Y., Wang Y., Zhao Y. (2023). The coexistence of bacterial species restructures biofilm architecture and increases tolerance to antimicrobial agents. Microbiol. Spectr..

[7] Krzyściak W., Jurczak A., Kościelniak D., Bystrowska B., Skalniak A. (2014). The virulence of *Streptococcus mutans* and the ability to form biofilms. Eur. J. Clin. Microbiol. Infect. Dis..

[8] Kara D., Luppens S.B., van Marle J., Ozok R., ten Cate J.M. (2007). Microstructural differences between single-species and dual-species biofilms of *Streptococcus mutans* and *Veillonella parvula*, before and after exposure to chlorhexidine. FEMS Microbiol. Lett..

[9] Luppens S.B., Kara D., Bandounas L., Jonker M.J., Wittink F.R., Bruning O., Breit T.M., Ten Cate J.M., Crielaard W. (2008). Effect of *Veillonella parvula* on the antimicrobial resistance and gene expression of *Streptococcus mutans* grown in dual-species biofilm. Oral Microbiol. Immunol..

[10] Khoury Z.H., Vila T., Puthran T.R., Sultan A.S., Montelongo-Jauregui D., Melo M.A.S., Jabra-Rizk M.A. (2020). The Role of Candida albicans Secreted Polysaccharides in Augmenting *Streptococcus mutans* Adherence and Mixed Biofilm Formation: In vitro and in vivo Studies. Front. Microbiol..

[11] Li Y., Huang S., Du J., Wu M., Huang X. (2023). Current and prospective therapeutic strategies: Tackling *Candida albicans* and *Streptococcus mutans* cross-kingdom biofilm. Front. Cell. Infect. Microbiol..

[12] Pathak J.L., Yan Y., Zhang Q., Wang L., Ge L. (2021). The role of oral microbiome in respiratory health and diseases. Respir. Med..

[13] Chu X.J., Cao N.W., Zhou H.Y., Meng X., Guo B., Zhang H.Y., Li B.Z. (2021). The oral and gut microbiome in rheumatoid arthritis patients: A systematic review. Rheumatology.

[14] Koliarakis I., Messaritakis I., Nikolouzakis T.K., Hamilos G., Souglakos J., Tsiaoussis J. (2019). Oral Bacteria and Intestinal Dysbiosis in Colorectal Cancer. Int. J. Mol. Sci..

[15] Wang N., Fang J.Y. (2023). Fusobacterium nucleatum, a key pathogenic factor and microbial biomarker for colorectal cancer. Trends Microbiol..

[16] Sansores-España D., Carrillo-Avila A., Melgar-Rodriguez S., Díaz-Zuñiga J., Martínez-Aguilar V. (2021). Periodontitis and Alzheimer’s disease. Med. Oral Patol. Oral Cir. Bucal.

[17] Zhang Z., Liu D., Liu S., Zhang S., Pan Y. (2021). The Role of *Porphyromonas gingivalis* Outer Membrane Vesicles in Periodontal Disease and Related Systemic Diseases. Front. Cell. Infect. Microbiol..

[18] Rabe L.K., Winterscheid K.K., Hillier S.L. (1988). Association of viridans group streptococci from pregnant women with bacterial vaginosis and upper genital tract infection. J. Clin. Microbiol..

[19] Cassini M.A., Pilloni A., Condò S.G., Vitali L.A., Pasquantonio G., Cerroni L. (2013). Periodontal bacteria in the genital tract: Are they related to adverse pregnancy outcome?. Int. J. Immunopathol. Pharmacol..

[20] Ishihara K., Okuda K. (1999). Molecular pathogenesis of the cell surface proteins and lipids from Treponema denticola. FEMS Microbiol. Lett..

[21] Cabacungan E., Pieringer R.A. (1980). Excretion of extracellular lipids by *Streptococcus mutans* BHT and FA-1. Infect. Immun..

[22] Sallans L., Giner J.L., Kiemle D.J., Custer J.E., Kaneshiro E.S. (2013). Structural identities of four glycosylated lipids in the oral bacterium *Streptococcus mutans* UA159. Biochim. Biophys. Acta.

[23] Custer J.E., Goddard B.D., Matter S.F., Kaneshiro E.S. (2014). The relative proportions of different lipid classes and their fatty acid compositions change with culture age in the cariogenic dental pathogen *Streptococcus mutans* UA159. Lipids.

[24] Lattif A.A., Mukherjee P.K., Chandra J., Roth M.R., Welti R., Rouabhia M., Ghannoum M.A. (2011). Lipidomics of *Candida albicans* biofilms reveals phase-dependent production of phospholipid molecular classes and role for lipid rafts in biofilm formation. Microbiology.

[25] Hans S., Fatima Z., Hameed S. (2022). Mass spectrometry-based untargeted lipidomics reveals new compositional insights into membrane dynamics of *Candida albicans* under magnesium deprivation. J. Appl. Microbiol..

[26] Wood P.L., Erol E. (2023). Construction of a Bacterial Lipidomics Analytical Platform: Pilot Validation with Bovine Paratuberculosis Serum. Metabolites.

[27] Klatt S., Brammananth R., O’Callaghan S., Kouremenos K.A., Tull D., Crellin P.K., Coppel R.L., McConville M.J. (2018). Identification of novel lipid modifications and intermembrane dynamics in *Corynebacterium glutamicum* using high-resolution mass spectrometry. J. Lipid Res..

[28] Wood P.L. (2017). Non-targeted lipidomics utilizing constant infusion high resolution ESI mass spectrometry. Springer Protocols, Neuromethods: Lipidomics.

[29] Wood P.L., Woltjer R.L., Wood P.L. (2021). Electrospray Ionization High Resolution Mass Spectrometry of the Chloride Adducts of Steroids, Mono- and Oligo-saccharides, Xyloglucans, Ceramides, Gangliosides, and Phenols. Springer Protocols, Neuromethods: Metabolomics.

[30] Chwastek G., Surma M.A., Rizk S., Grosser D., Lavrynenko O., Rucińska M., Jambor H., Sáenz J. (2020). Principles of Membrane Adaptation Revealed through Environmentally Induced Bacterial Lipidome Remodeling. Cell Rep..

[31] Frantsuzova E., Bogun A., Vetrova A., Delegan Y. (2022). Methods of Identifying Gordonia Strains in Clinical Samples. Pathogens.

[32] Cohen L.J., Kang H.S., Chu J., Huang Y.H., Gordon E.A., Reddy B.V., Ternei M.A., Craig J.W., Brady S.F. (2015). Functional metagenomic discovery of bacterial effectors in the human microbiome and isolation of commendamide, a GPCR G2A/132 agonist. Proc. Natl. Acad. Sci. USA.

[33] Cohen L.J., Esterhazy D., Kim S.H., Lemetre C., Aguilar R.R., Gordon E.A., Pickard A.J., Cross J.R., Emiliano A.B., Han S.M. (2017). Commensal bacteria make GPCR ligands that mimic human signalling molecules. Nature.

[34] Lynch A., Tammireddy S.R., Doherty M.K., Whitfield P.D., Clarke D.J. (2019). The Glycine Lipids of Bacteroides thetaiotaomicron are Important for Fitness during Growth in Vivo and in Vitro. Appl. Environ. Microbiol..

[35] Wood P.L. (2020). Fatty Acyl Esters of Hydroxy Fatty Acid (FAHFA) Lipid Families. Metabolites.

[36] Brejchova K., Balas L., Paluchova V., Brezinova M., Durand T., Kuda O. (2020). Understanding FAHFAs: From structure to metabolic regulation. Prog. Lipid Res..

[37] Riecan M., Paluchova V., Lopes M., Brejchova K., Kuda O. (2022). Branched and linear fatty acid esters of hydroxy fatty acids (FAHFA) relevant to human health. Pharmacol. Ther..

[38] Butovich I.A. (2013). Tear film lipids. Exp. Eye Res..

[39] Yasuda S., Okahashi N., Tsugawa H., Ogata Y., Ikeda K., Suda W., Arai H., Hattori M., Arita M. (2020). Elucidation of Gut Microbiota-Associated Lipids Using LC-MS/MS and 16S rRNA Sequence Analyses. iScience.

[40] Wood P.L., Scoggin K., Ball B.A., Lawrence L., Troedsson M.H., Squires E.L. (2016). Lipidomics of equine sperm and seminal plasma: Identification of amphiphilic (O-acyl)-ω-hydroxy-fatty acids. Theriogenology.

[41] Wood P.L., Ball B.A., Scoggin K., Troedsson M.H., Squires E.L. (2018). Lipidomics of equine amniotic fluid: Identification of amphiphilic (O-acyl)-ω-hydroxy-fatty acids. Theriogenology.

[42] Dietz C., Clark R.B., Nichols F.C., Smith M.B. (2017). Convergent synthesis of a deuterium-labeled serine dipeptide lipid for analysis of biological samples. J. Label. Comp. Radiopharm..

[43] Farrokhi V., Nemati R., Nichols F.C., Yao X., Anstadt E., Fujiwara M., Grady J., Wakefield D., Castro W., Donaldson J. (2013). Bacterial lipodipeptide, Lipid 654, is a microbiome-associated biomarker for multiple sclerosis. Clin. Transl. Immunol..

[44] Nemati R., Dietz C., Anstadt E.J., Cervantes J., Liu Y., Dewhirst F.E., Clark R.B., Finegold S., Gallagher J.J., Smith M.B. (2017). Deposition and hydrolysis of serine dipeptide lipids of Bacteroidetes bacteria in human arteries: Relationship to atherosclerosis. J. Lipid Res..

[45] Nichols F.C., Clark R.B., Liu Y., Provatas A.A., Dietz C.J., Zhu Q., Wang Y.H., Smith M.B. (2020). Glycine Lipids of *Porphyromonas gingivalis* are Agonists for Toll-like Receptor 2. Infect. Immun..

[46] Nichols F.C., Clark R.B., Maciejewski M.W., Provatas A.A., Balsbaugh J.L., Dewhirst F.E., Smith M.B., Rahmlow A. (2020). A novel phosphoglycerol serine-glycine lipodipeptide of *Porphyromonas gingivalis* is a TLR2 ligand. J. Lipid Res..

[47] Nichols F.C., Bhuse K., Clark R.B., Provatas A.A., Carrington E., Wang Y.H., Zhu Q., Davey M.E., Dewhirst F.E. (2021). Serine/Glycine Lipid Recovery in Lipid Extracts from Healthy and Diseased Dental Samples: Relationship to Chronic Periodontitis. Front. Oral Health.

[48] Olsen I., Nichols F.C. (2018). Are Sphingolipids and Serine Dipeptide Lipids Underestimated Virulence Factors of Porphyromonas gingivalis?. Infect. Immun..

[49] Farrugia C., Stafford G.P., Murdoch C. (2020). *Porphyromonas gingivalis* Outer Membrane Vesicles Increase Vascular Permeability. J. Dent. Res..

[50] Brown J., Everett C., Barragan J.A., Vargas-Medrano J., Gadad B.S., Nichols F., Cervantes J.L. (2022). Multiple Sclerosis-associated Bacterial Ligand 654. Arch. Med. Res..

[51] Heidler von Heilborn D., Nover L.L., Weber M., Hölzl G., Gisch N., Waldhans C., Mittler M., Kreyenschmidt J., Woehle C., Hüttel B. (2022). Polar lipid characterization and description of *Chryseobacterium capnotolerans* sp. nov., isolated from high CO_2_-containing atmosphere and emended descriptions of the genus *Chryseobacterium*, and the species *C. balustinum*, *C. daecheongense*, *C. formosense*, *C. gleum*, *C. indologenes*, *C. joostei*, *C. scophthalmum* and *C. ureilyticum*. Int. J. Syst. Evol. Microbiol..

[52] Bill M.K., Brinkmann S., Oberpaul M., Patras M.A., Leis B., Marner M., Maitre M.P., Hammann P.E., Vilcinskas A., Schuler S.M.M. (2021). Novel Glycerophospholipid, Lipo- and N-acyl Amino Acids from Bacteroidetes: Isolation, Structure Elucidation and Bioactivity. Molecules.

[53] Sartorio M.G., Valguarnera E., Hsu F.F., Feldman M.F. (2022). Lipidomics Analysis of Outer Membrane Vesicles and Elucidation of the Inositol Phosphoceramide Biosynthetic Pathway in *Bacteroides thetaiotaomicron*. Microbiol. Spectr..

[54] Kawasaki K., Gomi K., Kawai Y., Shiozaki M., Nishijima M. (2003). Molecular basis for lipopolysaccharide mimetic action of Taxol and flavolipin. J. Endotoxin Res..

[55] Ryan E., Joyce S.A., Clarke D.J. (2023). Membrane lipids from gut microbiome-associated bacteria as structural and signalling molecules. Microbiology.

[56] Nichols F.C. (1994). Distribution of 3-hydroxy iC17:0 in subgingival plaque and gingival tissue samples: Relationship to adult periodontitis. Infect. Immun..

[57] Guan Z., Chen L., Gerritsen J., Smidt H., Goldfine H. (2016). The cellular lipids of Romboutsia. Biochim. Biophys. Acta..

[58] Guan Z., Garrett T.A., Goldfine H. (2019). Lipidomic Analysis of *Clostridium cadaveris* and *Clostridium fallax*. Lipids.

[59] Guan Z., Goldfine H. (2021). Lipid diversity in clostridia. Biochim. Biophys. Acta Mol. Cell Biol. Lipids.

[60] Lopes C., Barbosa J., Maciel E., da Costa E., Alves E., Domingues P., Mendo S., Domingues M.R.M. (2019). Lipidomic signature of Bacillus licheniformis I89 during the different growth phases unravelled by high-resolution liquid chromatography-mass spectrometry. Arch. Biochem. Biophys..

[61] Wei Y., Joyce L.R., Wall A.M., Guan Z., Palmer K.L. (2021). Streptococcus pneumoniae, *S. mitis*, and *S. oralis* Produce a Phosphatidylglycerol-Dependent, *ltaS*-Independent Glycerophosphate-Linked Glycolipid. mSphere.

[62] Nakano M., Fischer W. (1978). Trihexosyldiacylglycerol and acyltrihexosyldiacylglycerol as lipid anchors of the lipoteichoic acid of Lactobacillus casei DSM 20021. Biol. Chem..

[63] Shiraishi T., Yokota S., Morita N., Fukiya S., Tomita S., Tanaka N., Okada S., Yokota A. (2013). Characterization of a *Lactobacillus gasseri* JCM 1131T lipoteichoic acid with a novel glycolipid anchor structure. Appl. Environ. Microbiol..

[64] Shiraishi T., Yokota S., Sato Y., Ito T., Fukiya S., Yamamoto S., Sato T., Yokota A. (2018). Lipoteichoic acids are embedded in cell walls during logarithmic phase, but exposed on membrane vesicles in *Lactobacillus gasseri* JCM 1131^T^. Benef. Microbes.

[65] Shiraishi T., Yamamoto S., Yokota S.I. (2019). Structural analysis of the lipoteichoic acid anchor glycolipid: Comparison of methods for degradation of the glycerophosphate backbone polymer. J. Microbiol. Methods.

[66] Nichols F.C., Riep B., Mun J., Morton M.D., Bojarski M.T., Dewhirst F.E., Smith M.B. (2004). Structures and biological activity of phosphorylated dihydroceramides of *Porphyromonas gingivalis*. J. Lipid Res..

[67] Dong P.T., Tian J., Kobayashi-Kirschvink K.J., Cen L., McLean J.S., Bor B., Shi W., He X. (2023). Episymbiotic bacterium induces intracellular lipid droplet production in its host bacteria. bioRxiv.

[68] Dewhirst F.E., Chen T., Izard J., Paster B.J., Tanner A.C., Yu W.H., Lakshmanan A., Wade W.G. (2010). The human oral microbiome. J. Bacteriol..

[69] Lazarevic V., Whiteson K., Hernandez D., François P., Schrenzel J. (2010). Study of inter- and intra-individual variations in the salivary microbiota. BMC Genom..

[70] Baronio M., Lattanzio V.M., Vaisman N., Oren A., Corcelli A. (2010). The acylhalocapnines of halophilic bacteria: Structural details of unusual sulfonate sphingoids. J. Lipid Res..

[71] Nichols F.C., Yao X., Bajrami B., Downes J., Finegold S.M., Knee E., Gallagher J.J., Housley W.J., Clark R.B. (2011). Phosphorylated dihydroceramides from common human bacteria are recovered in human tissues. PLoS ONE.

[72] Yin W., Wang Y., Liu L., He J. (2019). Biofilms: The Microbial "Protective Clothing" in Extreme Environments. Int. J. Mol. Sci..

[73] Yoo B.H., Kim. M.D. (2015). Isolation of the Inositol Phosphoceramide Synthase Gene (AUR1) from Stress-Tolerant Yeast Pichia kudriavzevii. J. Microbiol. Biotechnol..

[74] Panevska A., Skočaj M., Križaj I., Maček P., Sepčić K. (2019). Ceramide phosphoethanolamine, an enigmatic cellular membrane sphingolipid. Biochim. Biophys. Acta Biomembr..

[75] LaBach J.P., White D.C. (1969). Identification of ceramide phosphorylethanolamine and ceramide phosphorylglycerol in the lipids of an anaerobic bacterium. J. Lipid Res..

[76] Cerbón J., Falcon A., Hernández-Luna C., Segura-Cobos D. (2005). Inositol phosphoceramide synthase is a regulator of intracellular levels of diacylglycerol and ceramide during the G1 to S transition in *Saccharomyces cerevisiae*. Biochem. J..

[77] Grundner M., Munjaković H., Tori T., Sepčić K., Gašperšič R., Oblak Č., Seme K., Guella G., Trenti F., Skočaj M. (2022). Ceramide Phosphoethanolamine as a Possible Marker of Periodontal Disease. Membranes.

[78] Buré C., Cacas J.L., Mongrand S., Schmitter J.M. (2014). Characterization of glycosyl inositol phosphoryl ceramides from plants and fungi by mass spectrometry. Anal. Bioanal. Chem..

[79] Chaudhari P.N., Wani K.S., Chaudhari B.L., Chincholkar S.B. (2009). Characteristics of sulfobacin A from a soil isolate *Chryseobacterium gleum*. Appl. Biochem. Biotechnol..

[80] Tropis M., Meniche X., Wolf A., Gebhardt H., Strelkov S., Chami M., Schomburg D., Krämer R., Morbach S., Daffé M. (2005). The crucial role of trehalose and structurally related oligosaccharides in the biosynthesis and transfer of mycolic acids in Corynebacterineae. J. Biol. Chem..

[81] Guan Z., Katzianer D., Zhu J., Goldfine H. (2014). *Clostridium difficile* contains plasmalogen species of phospholipids and glycolipids. Biochim. Biophys. Acta.

[82] Walker A., Pfitzner B., Harir M., Schaubeck M., Calasan J., Heinzmann S.S., Turaev D., Rattei T., Endesfelder D., Castell W.Z. (2017). Sulfonolipids as novel metabolite markers of *Alistipes* and *Odoribacter* affected by high-fat diets. Sci. Rep..

[83] Godchaux W., Leadbetter E.R. (1984). Sulfonolipids of gliding bacteria. Structure of the N-acylaminosulfonates. J. Biol. Chem..

[84] Schorey J.S., Sweet L. (2015). The mycobacterial glycopeptidolipids: Structure, function, and their role in pathogenesis. Mol. Microbiol..

[85] Ooshima T., Kuramitsu H.K. (1981). Regulation of extracellular slime production by *Actinomyces viscosus*. Infect. Immun..

[86] Zvanych R., Lukenda N., Li X., Kim J.J., Tharmarajah S., Magarvey N.A. (2015). Systems biosynthesis of secondary metabolic pathways within the oral human microbiome member *Streptococcus mutans*. Mol. Biosyst..

[87] Cañavate J.P., Armada I., Ríos J.L., Hachero-Cruzado I. (2016). Exploring occurrence and molecular diversity of betaine lipids across taxonomy of marine microalgae. Phytochemistry.

[88] Wood P.L., Wood M.D., Kunigelis S.C. (2023). Pilot Lipidomics Study of Copepods: Investigation of Potential Lipid-Based Biomarkers for the Early Detection and Quantification of the Biological Effects of Climate Change on the Oceanic Food Chain. Life.

[89] Tsugawa H., Satoh A., Uchino H., Cajka T., Arita M., Arita M. (2019). Mass Spectrometry Data Repository Enhances Novel Metabolite Discoveries with Advances in Computational Metabolomics. Metabolites.

[90] Reddy M.M., Goossens C., Zhou Y., Chaib S., Raviglione S., Nicolè F., Hume B.C.C., Forcioli D., Agostini S., Boissin E. (2023). Multi-omics determination of metabolome diversity in natural coral populations in the Pacific Ocean. Commun. Earth Environ..

[91] Angelini R., Babudri F., Lobasso S., Corcelli A. (2010). MALDI-TOF/MS analysis of archaebacterial lipids in lyophilized membranes dry-mixed with 9-aminoacridine. J. Lipid Res..

